# Application of ultrasound technology for the effective management of waste from fruit and vegetable

**DOI:** 10.1016/j.ultsonch.2023.106744

**Published:** 2023-12-29

**Authors:** Brera Ghulam Nabi, Kinza Mukhtar, Sadia Ansar, Syed Ali Hassan, Muhammad Adnan Hafeez, Zuhaib F. Bhat, Amin Mousavi Khaneghah, Ahsan Ul Haq, Rana Muhammad Aadil

**Affiliations:** a National Institute of Food Science and Technology, University of Agriculture, Faisalabad 38000, Pakistan; b Department of Human Nutrition and Food Technology, Faculty of Allied Health Sciences, Superior University Lahore, Pakistan; c Division of Livestock Products Technology, Skuast-J, Jammu, India; d Department of Fruit and Vegetable Product Technology, Institute of Agricultural and Food Biotechnology – State Research Institute, Warsaw, Poland; e Department of Forestry & Range Management, Faculty of Agriculture, University of Agriculture, Faisalabad 38000, Pakistan; f Food Health Research Center, Hormozgan University of Medical Sciences, Bandar Abbas, Iran

**Keywords:** Fruits and vegetable waste, Sustainable food, Waste valorization, Upcycling waste, Perishable commodities, Ultrasound-assisted extraction

## Abstract

Food waste presents a continuous challenge for the food industry, leading to environmental pollution and economic issues. A substantial amount of waste, including by-products from fruits and vegetables, non-edible food items, and other waste materials, is produced throughout the food supply chain, from production to consumption. Recycling and valorizing waste from perishable goods is emerging as a key multidisciplinary approach within the circular bio-economy framework. This waste, rich in raw by-products, can be repurposed as a natural source of ingredients. Researchers increasingly focus on biomass valorization to extract and use components that add significant value. Traditional methods for extracting these bio-compounds typically require the use of solvents and are time-consuming, underscoring the need for innovative techniques like ultrasound (US) extraction. Wastes from the processing of fruits and vegetables in the food industry can be used to develop functional foods and edible coatings, offering protection against various environmental factors. This comprehensive review paper discusses the valorization of waste from perishable items like fruits and vegetables using US technology, not only to extract valuable components from waste but also to treat wastewater in the beverage industry. It also covers the application of biomolecules recovered from this process in the development of functional foods and packaging.

## Graphical abstract

**Figure:**
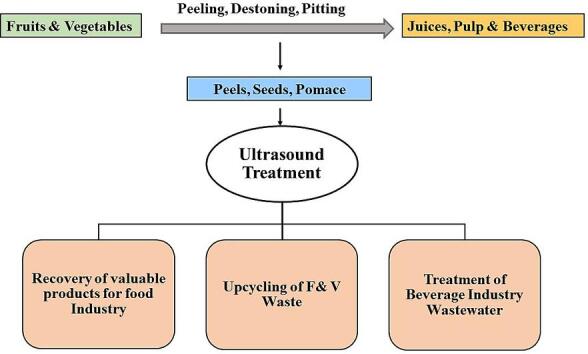

## 1 Introduction

Globally, the linear economic approach, which emphasizes responsible consumption and production with minimal waste accumulation, is predominant. Certain geographical regions experience higher food production rates due to favorable climatic conditions. However, these increased production rates also result in substantial waste materials. The vast amounts of waste are discarded in landfills, and only 12 % is recycled [1]. A circular economic approach is based on consumption and minimum waste production through the recycling and reusing waste components in new products. In this way, waste becomes an important resource for sustainability. There are two major international challenges; the first one is to provide food in surplus as per increased population demands and to overcome food shortage (food security), and the second one is a huge accumulation of food waste materials that has an impact on carbon footprints of the environment (food waste management) [2]. These two global issues trigger alterations in production systems to make them more sustainable and environmentally friendly. The circular economy is most appealing due to its major role in sustainable development, improved environment quality, and economic prosperity [3]. Fruits and vegetables produce large amounts of waste, which includes stems, leaves, peels, and seeds [4]. According to Suri et al., [5] more than 20 % of fruits and vegetables are wasted due to insufficient processing and management. For example, in kiwi production, the percentile is ∼ 1 × 106 tons of global waste annually [6]. In addition, growing interest in citrus fruit consumption increases waste production, which generates 50 % of the waste of fresh fruit [7]. Fruits and vegetables by-products comprise a wide variety of bioactive compounds. As bioactive compounds are biomass's main component, their waste is interesting for the bio-economy model and has a major role in the pharmaceutical, cosmetics, and food sectors. Waste streams such as seeds and peels also contain many essential oils enriched with bioactive compounds. They can sometimes be used directly as value-added products via various techniques [8], [9].

The valorization method offers a sustainable way to transform food waste into value-added products. However, improper treatment of this waste can lead to serious environmental issues, such as unpleasant odors and pollution. Valorizing by-products from fruits and vegetables is an effective strategy to not only reduce environmental waste but also to utilize valuable components from the waste, reintegrating them into the economy. This approach addresses both economic and environmental concerns. For example, using waste by-products not only conserves natural resources but also reduces landfill costs. Moreover, it fosters innovation in the development of functional foods and packaging materials [10]. Food packaging is a tool utilized to protect food from environmental stress, such as light, heat, oxygen, and preservation of nutrients.

Moreover, consumer demand for natural ingredients in food products has increased due to health-related issues with synthetic products. The overall approach is significant when green technologies are applied to reduce hazardous compound production and decrease the severe environmental and health impact. Due to these reasons, the extraction, characterization, and purification of natural bioactive compounds have increased scientists' interest in using novel technologies to recover bioactive substances from food waste streams to make functional foods [11]. In addition, industrial processing water has become a serious concern due to bioactive compounds disposed of in wastewater. It is necessary to recover these bioactive compounds due to their role in developing functional foods and to lessen bad environmental impacts. Various technologies such as pulsed electric field (PEF), high-pressure processing [12], microwave (MW), and US [13] have been utilized for this purpose. In the following sections, the US is explored as a green technology for recovering valuable components from fruit and vegetable waste and the wastewater of these industries. Besides, this review focuses on the latest fruit and vegetable waste applications in functional foods development and for edible packaging to protect food from external issues.

## 2 Ultrasound

**Fig. 1:**
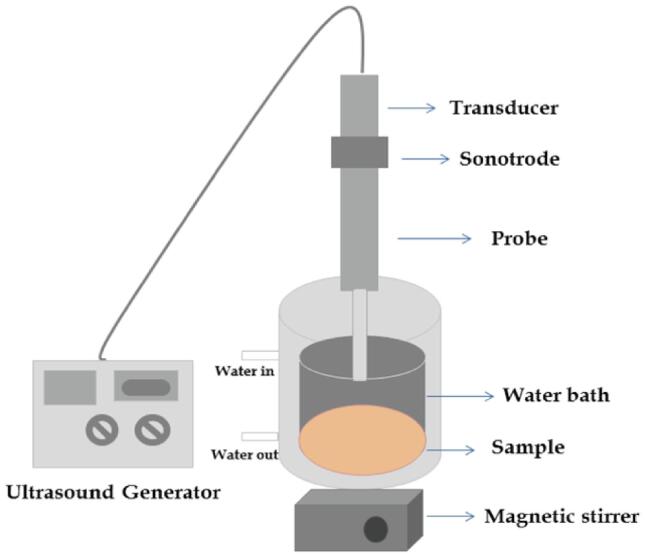
Schematic illustration of UAE.

The US is a green and environment-friendly technique with applications in various food industry processes. This technique is a great alternative to many heat and conventional treatments that can affect the quality of the product. Ultrasound-assisted extraction (UAE) is commonly employed in wave mode for short-term and pulse mode for long-term extraction. Its application in extraction is found to be effective and overcomes the hurdles of conventional techniques with improved yields of constituents [14], [15]. In this phenomenon, the implosion and cavitation cause rupture in cell walls and improve the mass transfer from solid to liquid state. This creates micro-channels within tissues, thus improving solvent penetration and enhancing mass transfer [16]. A US probe system is preferred over a US bath for extraction purposes due to the more powerful intensity delivered through a small surface in the extraction process. Probes are operated at 20 kHz and connected to a transducer, directly delivering US waves to extraction media with less ultrasonic energy loss [17]. Fig. 1 illustrates the UAE's setup for the food industry.

This is applied to extract bioactive compounds due to its adaptability, simple operation, less solvent requirement, and biological activity [18], [19]. The US has been applied to recover the polyphenols and other natural components from various fruits and vegetable by-products by utilizing hydroethanolic mixtures as solvents. For example, it showed more efficiency in extracting bioactive components from lemon waste using water as a solvent [20], [21]. The UAE has also attracted scientists for protein extraction [22], [23]. The fruits and vegetable processing industry generates a large amount of wastewater containing various organic compounds, pointing to serious environmental concerns [24]. It is an effective method for removing toxic and hazardous organic components from wastewater. Its utilization in wastewater treatment helps remove toxic and resistant pollutants such as aromatics, surfactants, and dyes. This process involves the oxidative breakdown of resistant wastewater compounds [25].

US efficiency is linked to common factors like processing temperature, solvent nature, ultrasonic reactor type, frequency, and power [26]. It has advantages over conventional heating treatments, like less energy requirement, short processing time, less solvent consumption, and enhanced yield [27]. It has also proved effective for heat-sensitive compound extraction due to less temperature requirement [28]. Due to these capabilities, the US' involvement in waste extraction has become a recent trend worldwide.

## 3 Recovery of biomolecules from food waste

**Fig. 2:**
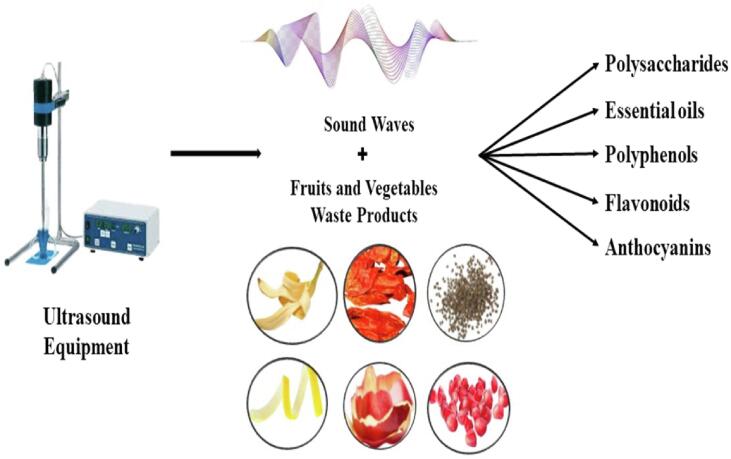
Recovery of valuable products from fruit and vegetable waste by US.

Fruits and vegetable wastes contain valuable compounds such as vitamins, carotenoids, alkaloids, proteins, phenolics, dietary fiber, and polyphenols. Fruit waste processing contains cellulose (40–50 %), lignin (10–25 %), hemicellulose (30 %), and other polysaccharide [29], [30]. Due to these reasons, there is a great chance of recovering natural components from these residues within the frame of the circular economy. The recovery of valuable products from fruit and vegetable waste using US technology is described in Fig. 2.

Fruit industries' waste is rich in nutritious ingredients compared to chemical and canning industries, which account for a lower content of flavonoids [31]. For instance, 60 % of the vitamin C is reported in acerola waste compared to fruit pulp [32]. It produced 40 % of by-products; seeds and peels contributed 30 % and 10 % of sludge during the juice clarification step that could extract vitamin C to fulfill the increased societal demand [33]. Besides, citrus industries produce approximately 50–60 % of waste and over 60 million tons globally [34]. For instance, grapefruit peel contributes 25–30 % to the whole grapefruit, while lemon peels give 12–13 % to the whole species. On the other hand, pomelo peel contributes 36–40 % of the fruit [35]. Moreover, the citrus peel is rich in pectin, limonene, and molasses [36]. Table 1 discusses the important studies of UAE of phytochemicals from fruit waste and byproducts.

**Table 1:** Ultrasound-assisted extraction for fruit waste valorization.

**Fruit source**	**Target compounds**	**Extraction conditions**	**Total yield**	**Reference**
Avocado peels	Bioactive compounds	Ethanol 38.46 %, 44.06 min, 50 °C, 37 kHz	TPC 45.34 mg GAE/g, TFC 87.56 mg RE/g, DPPH 73.25 mg TE/g, ABTS 160.34 mg TE/g, FRAP 44.65 mg TE/g.	[37]
Sichuan red orange peel	Tangeretin&nobiletin	Ethanol 85 %, LS 20:1 mL/g, 40 min, 50 °C, 150 W, 20 kHz	Tangeretin 2.6 mg/g,Nobiletin 6.4 mg/g.	[38]
Mandarin peel	Phenolic content	48 °C, 56.71 W, 40 min, 38.5 kHz	Total yield 26.52 %, TPC 15,263.32 mg eqgallic/100 g DW, Hesperidin 6,435.53 mg/100 g DW.	[39]
Mandarin peels	Pectin	80 °C, 37 kHz, 30 min	Pectin yield 21.3 ± 1.2 %.	[40]
Kinnowpomace	Functional compounds	Amplitude 40 %, LS 38:1, 40 °C, 12 min	TPC 7.89 ± 0.09 mg GAE/g, DPPH 68.63 ± 0.79 %, FRAC 31.17 ± 0.23 mM Fe^2+^/100 g, TDF 33.18–45.12 %.	[41]
Orange peel	Carotenoids	35 min, 42 °C, LS 15 mL/g	TCC 1.85 mg/100 g	[42]
Orange peels	Antioxidants	30 min, 60 °C, 15 mL/g	Gallic acid 157.08 mg/100 g DW, Ferulic acid 20.91 mg/100 g DW, IC50 65.5 μg/mL, TAC 2.79 g GAE/100 g DW.	[43]
Orange peel	Bioactive compounds	400 W, 30 min, Ethanol 50 %	TCC 0.63 mg β-carotene/100 g, Vitamin C 53.78 mg ascorbic acid/100 g, TPC 105.96 mg GAE/100 g, ORAC 27.08 mM TE, Hesperidin 113.03 ± 0.08 mg/100 g.	[44]
Rambutan peel	Anthocyanins	3 min, 20 kHz, Ethanol 19.5 %	Total anthocyanins 16.2 mg/g extract	[45]
Date palm seeds	Bioactive compounds	15 min, Amplitude 90 %, Solvents 70 %	TPC 145.54 ± 1.54 mg GAE/g powder, DDPH 719.19 ± 2.09 mol TE/g powder.	[46]
Aroniamelanocarpapomace	Anthocyanins	37 kHz, 70 °C, 200 W, 45 min	Total anthocyanins 88 %.	[47]
Orange peels	Polyphenols	UI 0.956 W/cm^2^, 59.83 °C, 25 kHz, 150 W	TPC 50.02 mg GA/100 g DM.	[48]
Kinnowpeel	Polyphenols	45 °C, 60 min, 35 kHz, 80 % methanol	TPC 32.48 mg gallic acid equivalent (GAE)/g extract.	[49]
Soursop fruit by-products	Phenolic compounds	5–15 min, Pulse cycle 0.4–1 s, Amplitude 40 %-100 %	TPC 187.32 mg/g DM, Columella 164.14 mg/g DM, seed 36.15 mg/g DM, pulp 33.24 mg/g DM, total yield 32–37 %, gallic acid 0.36–15.86 g/g DM, coumaric acid 0.07–1.37 g/g DM, chlorogenic acid 9.18–32.67 g/g.	[50]
Grape pomace	Phenolics	20–60 °C, Amplitude 20–60 %, LS 8–24 mL/g, 240 min	Total yield 48.76 mg GAE/g dry pomace.	[51]
Jabuticaba peel	Phenolics&anthocyanins	LS 46 %, 30 °C, 25 kHz, 150 W	TMA 4.8 mg/g dry peel, Gallic acid 92.8 mg/g dry peel, Cyanidin-3-O glucoside and 4.9 mg/g dry peel, Ellagic acid 7.8 mg/g dry peel.	[52]
Jabuticaba peel	Bioactive compounds	40 kHz, 150 W, 60 min, 30 °C	Anthocyanin 3.4 mg/g DW, TAC 841 μmol TE/g raw material.	[53]
Tomato, watermelon,& apple peel wastes	Proteins, Antioxidants, Cutin	98.6 W, amplitude 100 %, UI 6.48 W/cm^2^, 6 min	Maximum proteins 857 ± 1 mg BSA/g extract, TPC 107.2 ± 0.2 mg GAE/100 g DM in watermelon peel, Highest ABTS 1559 ± 20 μmol TE/100 g dm, FRAP 1767 ± 5 μmol TE/100 g dm, DDPH 902 ± 16 μmol in apple peel, tomato 42 % cutin, watermelon 55 %, cutin, apple 40 % cutin.	[54]
Grapefruit peel	Pectin	UI 12.56 W/cm^2^, 66.71 °C, 27.95 min, 800 W, 20 kHz	Yield 37.78 %.	[55]
Pomegranate peel	Pectin	20 kHz, 130 W, 29 min, 62 °C, LS 1:17.52 g/mL	Total yield 23.87 ± 0.28 %.	[56]
Pomegranate peel	Phenolic compounds	50 ± 1 °C, 15.12 min, amplitude 30 %, 20 kHz	Highest yield 42.45 %, TPC 354.67 mg GAE/g, total ellagitannin348.0 mg TAE/g, antioxidant activity 94.78 %, punicalagin 163.52 mg/g.	[57]
Pomegranate peels	Carotenoids	551.5 °C, LS 0.10, Amplitude 58.8 %	Total yield 0.33 mg carotenoids/100 g.	[58]
Jabuticabaepicarp	Anthocyanins	24.44 min, 500 W, 34.47 % ethanol	Total yield 31 ± 2 mg/g extract.	[59]
Pomelo peels	Pectin	2450 Hz & 40 kHz, 15 min, 360 W, LS 27 mL/g	Total yield 328.64 ± 4.19 mg/g.	[60]
Pomelo peels	Pectin	pH 1.80, 27.52 min, 643.44 W	Total yield 56.88 %.	[61]
Musa balbisiana waste	Pectin	323 w, pH 3.2, 27 min, LS 1:15 g/mL	Total yield 9.02 %.	[62]
Banana peel	Pectin	33.12 °C, 17.12 min, pH 3.68	Pectin yield 2.62 %, DOE 88.26 %, Galacturonic acid 88.26 %.	[63]
Jack fruit peel	Pectin	LS15:1 mL/g, pH 1.6, 24 min, 60 °C	Total yield 14.5 %.	[64]
*Citrus limetta* waste	d-limonene	60 °C, 80 W, 25 kHz, 20 min	Total yield 97 %, d-limonene 32.9 mg/g.	[65]
Orange waste	β-carotene	Pectinase 0.40 %, 115.55 min, pH 5.11	β-carotene content p ≤ 0.001.	[66]
Prickly pear peel	Bioactive compounds	2.5 min, LS 5 g/L, 30 °C, 40 ± 2 kHz	Betalains/g 201.6 mg, Phenolic acids 13.9 mg/g, Flavonoids 2.4 mg/g, Extractable solid 71.8 %, antioxidant activity 2.9.	[67]
Prickly pear peels & pulps	Colorants	400 W, amplitude 100 %, 24 kHz, 10 min	Total yield ≈50 mg/100 g FF.	[68]
Hybrid mandarin peels	Bioactive compounds	400 W, amplitude 80 %, 40 °C, 5–30 min	Maximum TFC 76.7 mg CE/100 g, ascorbic acid 136 mg ascorbic acid/100 g in ‘Clemenvilla’, TFC 1230 GAE/100 g, TCC 8173 μg β-carotene/100 g in ‘Ortanique’ and ‘Nadorcott’.	[69]
Papaya peel	Dietary fiber	NaOHconc 0.6 %-3.0 %, 30–80 °C, 10–60 min, LS ratio 10: 1–36: 1, 125–250 W	Total yield 36.99 %.	[70]
Peach waste	Antioxidant	Amplitude 23 %, 120 s, ethanol 0.70 g/g, 24 kHz, 900 W	TPC 309–317 mg GAE, TFC 94–120 mg QE, anthocyanins 8–9 mg CGE, ascorbic acid 2.1–2.2 mg TE.	[71]
Fig skin	Pectin	21.35 min, LS 24.66 mL/g, 70 °C, 70 W, 24 kHz	Total yield 14.0 %.	[72]
Olive waste	Phenolic compounds	40 min, 55 °C, 40 kHz, 200 W	Total yield 4.0 %, Hydroxytyrosol 17.2 mg/g, Tyrosol 14.1 mg/g.	[73]
Apple pomace	Pectin	20 kHz, 70 W, Amplitude 100 %, pH 1.8, LS 1:10 g/mL, 30 min	Total yield 9.183 %.	[74]
Apple pomace	Bioactive compounds	LS 1:10, 45 °C, 45 min	Total yield 58.09 %, DF 40.19 %, TPC 10.05 mg GAE/g, DDPH 10.50 mg TE/g, FRAP 1.27 mg TE/g, TMA 6.09 mg/L.	[75]
Apple pomace	Phenolic compounds	Solvent 1 %, 20 °C, 30 min, 50 ± 3 Hz, 300 W	Polyphenols 349.65 mg/dm^3^.	[76]
Banana bracts powder	Dietary fiber	LS 25 mL/g, Amplitude 49.48 %, 80 °C, 10 min	Total yield 71.05 %.	[77]
Mandarin epicarp flour	Carotenoids	42 kHz, 240 W, 60 °C, 60 min, LS 0.0004 g/mL	TCC 140.70 ± 2.66 mg β-carotene/100 g DW.	[78]
Sweet cherries skins	Anthocyanins Antioxidants	Ethanol 70 %, 40 kHz, 100 W, 40 °C, 30 min	Anthocyanins 14.48 ± 1.17 mg cyanidin 3-glucoside/100 g DW, ascorbic acid 85.37 ± 1.18 μMTrolox/100 g DW.	[79]
Passion fruit peel	Pectin	480 W, 65 °C, 15 min	Total yield 6.5 %.	[80]
Jackfruit pulp	Phenols and antioxidants	15 min, 250 W, LS 1:30, solvent 60 %	TPC 1.28 mg GAE/g (water extract), TPC 1.64 mg GAE/g (methanol extract), TPC 1.16 mg GAE/g (ethanol extract), TPC 1.57 mg GAE/g acetone extract, DDPH 28 % (water extract), DDPH 37 % (methanol extract), DDPH 27 % (ethanol extract), DDPH 25 % (acetone extract).	[81]
Jujube peels	Flavonoids	Ethanol 20 %, LS 1:30 g/mL, 200 W, 50 min	TFC 7.05 mg/g.	[82]
Mango by-products	Carotenoids	30 min, amplitude 30 %, 400 W, 24 kHz	β-cryptoxanthin 3.59 ± 0.43 mg/g DW, bioaccessibility β-cryptoxanthin 46.93 %, lutein 35.21 %, β-carotene 32.62 %.	[83]
Lime peel waste	Total phenolics	Ethanol 55 %, amplitude 38 %, 4 min	TPC 54.4 mg GAE/g.	[19]
Apricot pomace	Polyphenols	27 kHz, UI 6 W/cm^2^, 65 °C, 50 min	DPPH ∼ 70 %, ABTS ∼ 60 %, TPC 80.75 ± 1.85 mgAG/g, TFC 47.41 ± 1.20 mg QEs/g.	[84]
Guava leaves	Bioactive compounds	LS 0.02–0.2 g/mL, 5–20 min, 40–70 °C	TPC 15.5–68.8 mg GAE/g, antioxidant activity50.1–80.3 %, vitamin C 5.7–25.7 mg/100 g, TFC 44.7–289.77 mg QE/g.	[85]
Peach palm peels	Carotenoids	25 KHz, 25 °C, 5 min, Ethanol 1:10	TCC 67 mg/100 g.	[86]
Cactus fruit seed	Protein	25 ± 2 °C, 10 s, 450 W	Solubility 96.46 ± 2.07 %, Hydrophobicity 13.76 ± 0.85 μg, T-SH 50.25 ± 0.79 μmol/g, free-SH 8.60 ± 0.30 μmol/g.	[87]
Black jamun pulp	Phytocompounds	150 min, 70 °C, 40 kHz, 100 W	Anthocyanin 8.525 mg of C_3_Gg^−1^, TPC 47.331 mg GAE g^−1^, DPPH 80.17 %.	[88]
Tahiti lime by product	Bioactive compounds	480 W, 6 min, S/F 30	Total yield 5 %, TPC 7 mg GAE/g, hesperidin 0.85 mg/g DP, narirutin 0.15 mg/g DP TAC 50 mg TE/g DP.	[89]
Jabuticaba by products	Phenolic compounds	UI 3.7 W/cm^2^, 50 g/100 g, 19 kHz	TPC 3391 mg GAE/L, anthocyanin 287 mg/L, TFC 667 mg CE/L, tannins 11265 mg CE/L, FRAP 43600 μMol TE/L, ORAC 14578 μMol TE/L.	[90]
TCC: Total carotenoid content, FRAC: ferric reducing antioxidant capacity, TDF: total dietary fiber, TPC: total phenolic content, LS: Liquid-solid, TAC: Total antioxidant capacity, UI: Ultrasonic intensity, TMA: Total monomeric anthocyanin.				

Vegetable industrial processing such as carrots, tomatoes, and potatoes generate vast amounts of waste with beneficial bioactive compounds. For example, carrot industry waste is a rich source of pectin, α- and β-carotenes, lutein, and tocopherols associated with high antioxidant capacity [91]. Waste's main phenolic compounds are flavonoids such as quercetin and kaempferol glycosides [92]. Table 2 shows various studies of the valorization of vegetable waste in the US.

**Table 2:** Ultrasound for vegetable waste valorization.

**Food source**	**Bioactive compounds**	**Conditions**	**Yield**	**Reference**
Bottle gourd seeds	Dietary fiber & phytochemicals	LS23.90 mg/mL, 27.20 min, amplitude 47.76 %	Total dietary fiber 75.81 %, total yield phytochemicals 8.24 %, TPC 55.96 ± 0.75 mg/g, TFC 14.36 ± 0.45 mg/g, and antioxidant activity 35.08 ± 0.15 %.	[93]
Artichoke solid waste	Phenolic compounds	20 kHz, 240 W, ETOH 50 %, 10 min	Chlorogenic acid 97.86 %, TPC 220 mg gallic acid eq/g DW, DDPH 50 mg Troloxeq/g DW, FRAP 120 mg Troloxeq/g DW.	[94]
Lemon waste	Phenolic compounds	43 ± 2 kHz, 50 °C, 40 min, 200 W	p-coumaric acid 0.25 mg/g, Caffeic acids 0.58 mg/g, Hesperidin 6.59–7.84 mg/g.	[95]
Eggplant peel	Phenolic compounds	140 W, 37 kHz, 90 min, 50 °C, LS 100 mL/g	TPC 13.40–13.51 mg/g DW.	[96]
Eggplant peel	Phenolic compounds	1) 33.88 kHz, 69.4 °C 57.5 min, Methanol 76 % 2) 37 kHz, 55.1 °C, 44.85 min, Methanol 54 %	(1) TPC 29.63 g/100 g extract DW,(2) TA 2410.71 mg/100 g extract DW.	[97]
Eggplant peel	Phenolic compounds	400 W, 12 kHz, 30 min, LS 10:1	TPC 29.011 mg/g DW.	[98]
Eggplant peel	Anthocyanins	100 W, 40 kHz, 40 °C, LS 20 mL/g, Ethanol 70 %	Total anthocyanins 0.58 mg/g DW.	[99]
Eggplant peel	Pectin	50 W, 30 min, LS 20:1	Pectin 33.64 g/100 g.	[100]
Ginger stems and leaves powder	Polysaccharides	Dual 28&50, 28&68, 50&68 kHz, 30–50 min, 40–60 °C. Triple 28&50&68 kHz, 40–60 min, 40–60 °C.	Dual-frequency US 9.74 ± 0.30 %, triple-frequency US 10.50 ± 0.20 %.	[101]
Cantaloupe waste	Carotenoids	Amplitude 100 %, 10 min, LS 55 mL/g, 20 kHz	Lutein 63.24 ± 0.73 μg βCE/g DW, β-carotene 56.43 ± 0.11 μg βCE/g DW, TCC 124.61 ± 3.82 μg/g.	[102]
Bitter melon seeds	Protein	375 W, 20.0 min	Total yield 31.05 %.	[103]
Artichoke by products	Phenolic compounds	60 °C, 60 min, Ethanol 50 %, 37 kHz, 200 W	TPC 22.4 ± 0.2 mg/g DW, 3, 4-dicaffeoylquinic acid 32.8 ± 0.6 mg/g DW, chlorogenic acid 14.1 ± 0.2 mg/g DW.	[104]
Sugar beet pulp	Pectin	LS 44.03, 120.72 °C, 30.49 min	Total yield 24.63 %.	[105]
Red beet stalks	Natural pigments	53 °C, 89 W, 35 min, LS1:19 g/mL	Betacyanin 1.28 ± 0.02 mg/g, betaxanthin 5.31 ± 0.09 mg/g.	[106]
Red beetroot waste	Betalains& polyphenols	44 kHz, 30 min, 30 °C, 35 W, Ethanol 30 %	Betalain 7.7 % & 19.9 %, polyphenols 6.86 ± 0.23 mg/g DW.	[107]
Taro peel	Polysaccharide	Amplitude 50 %, 15 min, 20 kHz, 252.68 °C	Higher yield 3.65 g/100 g.	[108]
Eggplant peel	Bioactive compounds	45 kHz, 50–60 °C, 20–50 min	TPC 7.842 g/100 g, TMA 2,275 mg/kg, antioxidant activity 10.18 M Trolox/kg.	[109]
Carrot pomace	Carotenoids	17 min, 32 °C, Ethanol 51 %	TCC 31.82 ± 0.55.	[110]
Lettuce waste	Antioxidant Polyphenols	400 W, 24 kHz, 120 s	Total yield 81 μg/mL, antioxidant activity 101 μg TE/mL.	[111]
Tomato waste	Pectin	37 kHz, 15 min, 60 °C & 80 °C	Total yield 31.0 %-35.7 %.	[112]
Tomato peel	Pectin and polyphenols	15 min, 70 % ethanol, 400 W, 30 kHz, amplitude 95 %	TPC 1625.7 mg/100 g, pectin 3643.9 mg/100 g.	[113]
Tomato waste	Lycopene	45 min, 50 Hz, −40 °C	US: 45.51 ± 1.84 μg/g fresh wt, freeze drying: 104.10 ± 1.23 μg/g fresh wt, US + freeze drying: 138.82 ± 6.64 μg/g fresh wt.	[114]
Lemon waste	p-coumaric acid, caffeic acid, chlorogenic acid, hesperidin	10–60 min, 23–50 °C, 150–250 W, 43 ± 2 kHz	p-coumaric 0.25 mg/g, caffeic acids 0.58 mg/g, hesperidin 6.59–7.84 mg/g.	[95]
Black carrot pomace	Bioactive compounds	550 W, 37 kHz, 70 °C, 30 min	Total yield 0.08 kg/kg pomace, anthocyanins 297.9 mg/L Phenolics 1285.3 mg/L, antioxidant activity 37.6 μM/mL, DE 34.3 %.	[115]
Spinach extracts	Phenolic compound	37 kHz, 30 min, 40 °C, amplitude 50 %	TPC 64.88 ± 21.84 mg gallic acid/g DW, TFC 33.96 ± 11.30 mg/g DW, DPPH 64.18 ± 16.69 %, FRAC 70.25 ± 9.68 %.	[116]
Potato peels	Polyphenols	33 & 42 kHz, 30–45 °C	TPC 3.8–4.24 mg GAE gdb^-1^.	[117]
Potato by products	Phenolic compounds	35 min, 35 °C, LS1/10	Chlorogenic acid 49.3–61 %.	[118]

(TCC: Total carotenoid content, FRAC: ferric reducing antioxidant capacity, TDF: total dietary fiber, TPC: total phenolic content, LS: Liquid-solid, TAC: Total antioxidant capacity, UI: Ultrasonic intensity, TMA: Total monomeric anthocyanin).

### 3.1 Polysaccharides

Polysaccharides contain mainly cellulose, hemicellulose, and lignin; these components are made when three or more sugars are linked together in long chains and thus have complex structures [119]. The complicated structure with high lignocellulosic content makes further production complex and less effective. However, the US application transforms the complex polysaccharides into simple monomeric sugars and is then utilized for energy production [120]. In addition, it is a promising approach for lignin barrier disarrangement, hemicellulose, and cellulose crystal reduction. The removal of lignin and hemicellulose is related to the bond oxidation and amorphous nature [121], improving cellulose content [122], [123]. Polysaccharides are available in different plant tissues with the potential for anti-oxidation, anti-cancerous, and hypoglycemic levels. Their health benefits and less toxic nature make them beneficial ingredients for functional food development. Different plant parts not consumed by humans are subjected to the US for their extraction [124].

The Rambutan (*Nephelium lappaceum L.)* fruit peel was subjected to US for crude polysaccharides extraction. The outcomes demonstrated that US at the following conditions: power (110 W), temperature (53 °C), and processing time (41 min) resulted in the experimental yield of polysaccharides 8.29 % [125]. The papaya seed was subjected to alkaline and UAE alkaline extraction for soluble dietary fiber extraction, and the structure and compositions of the extracts obtained were compared. The US-treated samples at optimum conditions NaOH (0.6 %-3.0 %), temperature (30–80 °C), processing time (10–60 min), and power (125–250 W) resulted in the highest yield of dietary fibers (36.99 %). In addition, the US was combined with an alkaline treatment that showed fewer total amino acids but higher essential amino acids (16.18 %) than the simple alkaline treatment. The primary sugars were neutral in both treatments. The results indicated that papaya peel is a rich source of natural dietary fibers [70]. In addition another research, the US was used to extract polysaccharides from guava leaves by optimizing Box-Behnken design. The total yield of polysaccharides was 1.00 ± 0.04% at extraction time of 20 min, temperature of 62 ℃ and ultrasound power of 404 W. While the DPPH and ABTS⁺ radical scavenging rate of were 56.38% and 51.73%, respectively. The results indicated that guava leaves are rich source of polysaccharides [126].

Anwar et al. [108] compared the US with conventional and PEF-assisted extraction techniques to investigate the effects on yield and properties of water-soluble non-starch polysaccharides of taro peel. The finding showed that the US gave a higher yield (3.65 g/100 g) as compared to PEF-assisted extraction (2.25 g/100 g) and conventional extraction (2.10 g/100 g). The extracted starch sample after UAE treatment showed fewer impurities and lighter color, so it could be concluded that the US exhibits more advantages in extraction compared to other non-thermal and conventional methods [108]. The ginger stems' and leaves' polysaccharides were subjected to dual- and triple-frequency US extraction to assess physicochemical, structural, and biological activities. In dual frequency treatment, the following parameters were applied (dual-frequency: 28 & 50, 28 & 68, 50 & 68 kHz), (extraction time: 30, 40, 50 min), (extraction temperature: 40, 50, 60 ˚C), and (liquid-material ratio: 20, 25, 30 mL/g) to obtain the maximum yield. After that, triple frequency US involved 28, 50, and 68 kHz with time 40, 50, and 60 min, liquid–solid ratio 15, 20, and 25, and temperature 40, 50, 60 ◦C. The results revealed that triple-frequency US was more efficient in achieving higher polysaccharide yield (10.50 ± 0.20 %) than dual-frequency (9.74 ± 0.30 %). This higher yield was attributed to the higher collapsing of cavitation bubbles. In dual frequency, neutral sugars in samples were 52.14 ± 1.61 % while 58.81 ± 2.09 %, with triple frequency treatment. The uronic acid and protein content for dual frequency were 3.93 ± 0.20 % and 6.02 ± 0.24 %, while for triple frequency, it was 4.82 ± 0.08 % and 6.78 ± 0.11 %, respectively. Overall, these outcomes implied that the US, with triple frequency, improved the extraction of chemical moieties [101]. The US and alkaline solution were used for wampee seed protein extraction. The outcomes demonstrated that the optimum yield was 15.06 % at the LS ratio 1:29 g/mL, 64 min, and 12 pH [127]. Therefore, the final remarks stated that the US is a promising approach for polysaccharides extraction from fruit and vegetable waste at the industrial level.

### 3.2 Pectin

The presence of pectin in various plants' cell walls and middle lamella, including fruits and vegetables, has gained attention to exploring peel waste, pomace, and rind for the UAE. The fig skin was subjected to ultrasound-microwave (US-MW) assisted extraction at various conditions (10–30 min, 300–600 W, irradiation time 5–15 min, and LS ratio 10–30 mL/g) for extraction and obtained the highest pectin yield (13.97 %) [72]. Many studies have proven the UAE's efficiency in achieving a high yield of bioactive compounds from peel waste [95]. Various researchers evaluated the pectin extraction from different sources, but the citrus peel is a rich source of pectin compared to other fruit peels [128], [129]. Rhamnogalacturonan-I-enriched pectin was extracted from citrus peel and confirmed as having good thickening attributes compared to commercial pectin. In addition, it is reported as a suitable ingredient in functional food products due to its role in preventing cancer and heart diseases by rhamnogalacturonan-I-enriched pectin [130]. The US was applied for pectin extraction from sour orange peel. At the power 150 W, pH 1.5, and time of 10 min, optimum yield was obtained as 28.07 ± 0.67 %. While ash (1.89 ± 0.51), moisture (8.81 ± 0.68), and protein (1.45 ± 0.23 %) were improved, respectively. The maximum total phenolic content (TPC) (39.95 ± 3.13 mg gallic acid equivalents/g pectin), water holding (3.10 ± 0.12), oil holding capacity (1.32 ± 0.21 g), and degree of esterification (6.77 ± 0.43 %) were observed [131]. Apple pomace was subjected to the US at amplitude (20 %, 60 %, and 100 %), pH (1.5, 2, and 2.5), and time (10, 20, 30 min) for pectin extraction. The US amplitude greatly impacted the yield and degree of pectin esterification, while pH greatly influenced the yield of galacturonic acid and the degree of esterification. The optimum conditions were amplitude of 100 %, 1.8 pH, and 30 min treatment time, which resulted in a pectin yield of 9.183 %, galacturonic acid content of 98.127 g/100 g, and degree of esterification 83.202 % [74].

The UAE was applied at high intensity on mango peel for pectin extraction. The process was carried out at frequencies 37 kHz and 80 kHz, times 20 min, 25 min, and 30 min, and the ripening stages of the fruit were 0, 2, and 4. The findings showed that pectin yield ranged from 13 % to 30 % without time duration influence. The highest yield was obtained at the lowest frequency (37 kHz) and lowest maturity. Moreover, the lowest frequency (37 kHz) gave high gel strength, purity, and quality. The results demonstrated that the UAE application efficiently extracts pectin from mango waste [132]. Banana peel pectin was extracted by UAE application at optimum conditions temperature 33.12 °C, time 17.12 min, and pH 3.68. Under the following conditions, the maximum value was achieved (pectin yield 2.62 %, esterification degree 88.26 %, and galacturonic acid content 87 %). The results revealed that banana peel pectin was effectively extracted by US application [63]. Mono-sonication (pressure-assisted ultrasound technique) was applied for pectin extraction from various citrus cultivars in combination with US radiation and pressure. Applying optimum pressure during sonication enhances the yield of the target compound [133]. The UAE steam explosion with acid extraction and US steam explosion acid extraction were applied to passion fruit peel for pectin extraction. The pectin yields with UAE and acid extraction were 6.5 % and 5.3 %, respectively, but the emulsion stability in the former extraction method was poor. The US steam explosion acid extraction enhanced the emulsion stability in comparison to UAE and had more protein content (0.62 %). Moreover, it showed higher pectin extraction (10.7 %) and a low degree of esterification (68.5 %), but they exhibited lower thermal stability. The outcomes revealed that US steam explosion acid treatment was more effective and provided higher pectin yield [80].

Kazemi et al. [100] applied UAE for pectin extraction from eggplant byproducts. The findings showed a yield of 33.64 % at 50 W, but an increase in power from 50 W to 150 W negatively affected the pectin extraction as it degraded them. So, the US at optimum parameters affects the extraction yield positively. The US was applied at the following conditions: 550 W, 37 kHz, 70 °C /30 min on black carrot pomace for pectin extraction. The results showed the highest yield of pectin (0.08 kg/pomace), anthocyanins (297.9 mg/L), phenolics (1285.3 mg/L), and antioxidant activity (37.6 μM/mL). The overall findings indicated the presence of functional groups in pectin, so it can be concluded that US processing causes rapid extraction without adversely affecting its functionality [115]. Pectin and polyphenols were recovered from tomato peel waste by application of high hydrostatic pressure extraction (HHPE) and UAE. The HHPE increased by 15 % pectin recovery after 45 min time duration compared to traditional extraction for 180 min. Depectinized residues were treated with US for 15 min with 70 % ethanol, which gave twice lower TPC (1625.7 mg/100 g) than pectin samples (3643.9 mg/100 g) [113]. In conclusion, UAE is applied for pectin recovery from fruit and vegetable waste.

### 3.3 Essential oils

The UAE has the potential to enhance the recovery and yield of oils by adjusting processing parameters, including time, power, temperature, and frequency. Moreover, it can replace the harmful solvent utilized in chemical treatment [124]. It was applied to papaya seeds to evaluate the yield, antioxidant activity, and oil stability. The basic goal of this research was to obtain optimum US conditions for maximum yield of papaya seed oil with high stability and antioxidant activity. The US treatment resulted in 73 % oil recovery from papaya seed. The highest antioxidant capacity was obtained at high temperatures. The overall outcomes suggested optimum conditions: temperature 62.5 °C, power 700 W, processing time 38.5 min with solvent to sample ratio (∼7:1 v/w) [134]. The watermelon seeds were subjected to UAE for crude oil extraction at 25–75 %, 45–55 °C, and 20–40 min, respectively. The optimum conditions were power 65 %, temperature 52 °C, and processing time 36 min, resulting in the highest crude oil yield (108.62 mg-extract/g-DM). The US-extracted oil yield was lower than that of the Soxhlet method. However, antioxidant activity (35.84 %) and TPC (23.26 mg GAE/g extract) were higher as compared to the Soxhlet method, in which antioxidant activity and TPC were 28.70 %, 11.34 mg GAE/g extract [135].

Mango kernel oil was extracted with MW and in combination with US. The combined treatment gave better oil yields (96.67 ± 1.30) than MW alone (88.42 ± 1.36 %). The US-MW treatment showed a maximum increase in the recovery of mango kernel oil [136]. The US pretreatment was applied to citrus waste for essential oil extraction. The outcomes showed that maximum yield (33 %) was obtained at the following conditions: amplitude (52.7 %), processing time (15.7 min), and LS ratio (3.2/1). The increased yield in the case of the US is associated with mass transfer through cell wall breakage and cavities formation improved oil recovery [137]. The cranberry seeds were treated in the US for oil extraction. The objective of that research was to determine optimum conditions for oil extraction. The outcomes showed that optimal conditions were amplitude (95 %) and extraction time (11.38 min), which resulted in maximum extraction yields (22.55 ± 0.36 %) [138]. Hence, it has been proved that the UAE is an effective technology for recovering and enhancing essential oil from fruits and vegetable seeds.

### 3.4 Polyphenols, flavonoids, and anthocyanins

It has been reported that lime peel waste was subjected to MW and UAE for phenolic compound extraction. The outcomes suggested the optimum condition for MW (ethanol 55 %, power 140 W, times 45 s with eight repetitions) while for US (ethanol 55 %, amplitude 38 %, time 4 min). The results showed that the US was effective, resulted in maximum TPC (54.4 mg GAE/g), and saved 33 % time compared to MW extraction [19]. Mango peels were subjected to natural deep eutectic solvents (NADESs) and the US for antioxidant recovery. The optimum conditions were water content 20 %, duty cycle 50 %, density 2 W/cm^3^, LS ratio 30:1, particle size 0.3 mm, 30 min time, and lactic acid glucose 5:1. The NADES gave maximum TPC (69.85 mg GAE/g of MP), TFC (16.5 mg QE/g of MP), and DDPH (35.37 μg/mL). However, the US, in combination with NADES, provided 1.4 times higher polyphenols, 1.7 times more total flavonoids, and 1.9 times more antioxidant activity. Moreover, 50 % less time was observed, and a 25 % reduction in solvent consumption was observed compared to the batch method with 80 % ethanol [139]. The US technology was applied to jabuticaba peels to obtain high-value co-products. The US at 3.7 W/cm^2^ and 50 g water/100 g resulted in the best recovery of bioactive compounds. The maximum values detected for bioactive compounds were polyphenol (3391 mg GAE/L), anthocyanin (287 mg/L), flavonoids (667 mg CE/L), and tannins (11265 mg CE/L). Compared to other conventional techniques, the US approach is promising for phenolic compound recovery at low costs and with no hazards or environmental effects [90]. Jujube peel was subjected to US for flavonoid extraction at 200 W, 50 min, K2HPO4 35 % (ethanol 20 %), and LS ratio 30:1 g/mL. Rutin, quercetin 3-β-D-glucoside, and kaempferol-3-O-rutinoside were extracted with an overall yield of 95.55 %. Hence, the US application increased the flavonoid release [82]. Pomegranate peel phenolic compounds at 15.12 min, amplitude 30 %, and gave a maximum yield of 42.45 %, while others were recorded as TPC 354.67 mg GAE/g, total ellagitannin 348.0 mg TAE/g, and antioxidant activity 94.78 %. They were enhanced due to cavitation, cell wall disruption, particle size reduction, and increased mass transfer rate [57]. The US and MW were applied on prickly pear peels for bioactive compounds extraction at optimum conditions t = 2.5 and 1.4 min, LS = 5 and 5 g/L, metOH = 34.6 and 0 % of methanol, and T = 30 and 36.6 °C. The US gave maximum betalains/g (201.6 mg), phenolic acid (13.9 mg), and flavonoids (2.4 mg), while MW gave betalains/g (132.9 mg), phenolic acid (8.0 mg), and flavonoids (1.5 mg). This research suggested that the US is a better extraction technique for bioactive compound recovery [67]. Ferarsa et al. (2018) employed solid lipid extraction (SLE) (75 °C and pH 2.0, 60 min) and UAE (30 min) to extract the anthocyanins from eggplant peels. The results showed the yields of 23.101 mg gallic acid equivalent (GAE)/g and 29.011 mg GAE/g for SLE and UAE, respectively. The UAE was evaluated at 33 and 42 kHz for polyphenols extraction from two varieties of potato peel cream-skinned Lady Claire (LC) and pink-skinned Lady Rosetta (LR) that are usually utilized in snack food industries. Low frequency (33 KHz), high power (100 W) in comparison to high frequency (42 kHz) and low power (50 W) increased the TPC, chlorogenic acid, and DDPH significantly from 3.8 to 4.24 mg GAE gdb^-1^, 5.98 to 8.69 mg gdb^-1^ and 3.16 to 3.66 mg TE gdb^-1^ respectively. Low frequency is linked with acoustic cavitation, which results in higher phenolic yield and antioxidant activity. Overall, results suggested that the UAE is an efficient green technology for the extraction of valuable compounds from wastes [117]. Purple turnip peels were investigated as an antioxidant ingredient. The US at the following conditions were applied to the sample: methanol solvent (80 %), amplitude 65 %, time 15 min, and carried out in an ultrasonic bath. The outcomes showed that TPC (169.29 ± 6.89 mg GAE/g DW), TMAC (159.53 ± 10.82 mg CGE/L) while the TAC of extracts in CUPRAC assays was (44.19 ± 0.10 mg TE/g DW) and in DDPH assay (38.52 ± 0.06 mg TE/g DW). The results showed the bioactive compounds of purple turnip peel, which could be used in various food products [140].

Kinnow pomace, which is profuse with polyphenols, antioxidants, and dietary fiber, is discarded by processing industries. These compounds can be obtained from waste and have applications in food products as functional components. The US at the 40 % amplitude, 38:1 LS ratio, 40 °C temperature, and 12 min increased polyphenols (7.89 ± 0.09 mg GAE/g pomace), DPPH (68.63 ± 0.79 %), and FRAP (31.17 ± 0.23 mM Fe 2^+^ /100 g) was observed. The waste after polyphenol extraction also showed TDF (33.18–45.12 %), which included insoluble dietary fiber (68–72 %). This study can be scaled up for highly functional compound extraction from kinnow pomace [41]. Tahiti lime pomace was subjected to the US for phenolic extraction. The US was applied at 160 to 792 W power, 2 to 10 min, and ethanol was used as the solvent. These parameters' effects were investigated on yields, hesperidin, polyphenols, narirutin yields, and antioxidant capacity. The optimum conditions were 480 W and 6 min, which resulted in 5 % extraction yield, polyphenols (7 mg GAE/g BS), hesperidin, narirutin (0.85 mg/g DP, 0.15 mg/g DP), and antioxidant capacities (50 mg TE/g DP). This study suggested that the US could have effective applications in pomace utilization [89]. Da Rocha & Noreña [141] applied US at 250, 350, and 450 W for 5, 10, and 15 min on grape pomace for bioactive compounds extraction, and results reported 45 % anthocyanin on 10 min exposure time. Phenolic and anthocyanin were extracted from jabuticaba peels by US bath at 25 and 40 kHz. The outcomes showed maximum extraction at 25 kHz with 10 min sample exposure [142]. The UAE was applied for polyphenolic extraction from grape pomace. It was subjected to the sample at the following conditions: temperature (20–60 °C), amplitude (20–60 %), LS ratio (8–24 mL/g), and ethanol (0–100 %) was used as a solvent. The maximum extraction (48.76 mg GAE/g dry pomace) was achieved at 56 °C, 8 mL/g LS ratio, and 34 % amplitude within 20 min. The outcomes suggested that UAE applications resulted in purified and higher yields of valuable bioactive compounds [51]. Andrade et al. [47] determined the optimum pressure, intensity, solvent concentration, and solvent flow rate conditions in UAE for anthocyanin extraction from black chokeberry (*Aronia melanocarpa*) pomace. Citric acid was used as an extraction solvent. The best conditions were a temperature of 70 ˚C, 180 bar pressure, 1.5 % wt citric acid, and 200 W power in a US bath. These conditions resulted in 88 % weight extraction of anthocyanin in 45–45 min. It is also stated that the US is more efficient at the lowest temperatures. The effect of temperature was investigated on anthocyanin yield at optimum conditions. In addition, compound stability was evaluated to prove that the system could operate at 80 ˚C. The outcomes reported a 19 % increase in anthocyanin yield. The apple pomace with ethanol (50:50, v/v) in 10:1 LS ratio was subjected to an ultrasonic bath at 45 ˚C for 45 min for bioactive compounds recovery. Fresh pomace yield increased twice, from 7.12 % to 13.61 %, while UAE showed a further yield increase (from 21.64 to 58.09 %) in the case of freeze-dried pomace. The TPC for fresh pomace was under 0.72 mg GAE (gallic acid equivalents)/g, while the freeze-dried sample resulted in up to 10.05 mg GAE/g dry weight (DW) [75].

Moreover, food industrial waste peels (∼55 %), seeds (<5%), peduncles (<5%), and the remains of the pulp (∼35 %) of oranges, bananas, pears, and apples in variable proportions were pretreated with, air oven and US. The optimized extraction conditions for dried food waste were amplitude 69.7 %, temperature 53.43 ˚C, and time 12 min. The outcomes showed the highest yield (52.6 %), protein content (0.42 mg/g), TPC (116.42 mg GAE/g), and antioxidant activity (44.95 mg Trolox/g) [143]. Peach juice waste, either in a frozen or air-dried state, was evaluated by the UAE. The optimum conditions for frozen waste were UAE at 23 % amplitude and 120 s time. They resulted in lower polyphenols, flavonoids, anthocyanin contents, and antioxidant activity (309–317 mg GAE, 94–120 mg QE, 8–9 mg CGE, 2.1–2.2 mg TE, respectively) in the frozen waste. While polyphenols in dried waste were high (630–670 mg GAE), the flavonoids showed reduction (75–90 mg QE), while anthocyanin and vitamin C were non-significant in dried waste. This research suggested not only the extraction of bioactive compounds from peach waste but also the economic and environmental role of the US [71]. Guava leaf powder was subjected to normal and pulsed US for bioactive compound extraction. The findings revealed that normal US exhibited TPC (15.5–68.8 mg GAE/g), antioxidant activity (50.1–80.3 %), vitamin C (5.7–25.7 mg/100 g) and TFC (44.7–289.77 mg QE/g) with 5–20 min time and 40–70 °C temperature, while in pulsed US following conditions temperature (62.19 °C), extraction time (14.94 min) resulted maximum TPC (72.62 mg GAE/g), TFC (288.13 mg QE/g) and antioxidant activity (86.07 %). The outcomes suggested that the pulsed mode of the US was more efficient than the normal US [85]. Tomato, watermelon, and apple wastes were subjected to cascade extraction based on UAE. During the first extraction step, protein and antioxidants were achieved at NaOH 3 wt%, 98.6 W, 100 % amplitude, 6.48 W/cm^2^, and 6 min. The comparison revealed that watermelon peel had higher protein (857 ± 1 mg BSA/g extract) and TPC (107.2 ± 0.2 mg GAE/100 g DW) while the highest antioxidant activity was for apple peel for ABTS (1559 ± 20 μmol TE/100 g DW), FRAP (1767 ± 5 μmol TE/100 g DW), and DDPH (902 ± 16 mol TE/100 g DW). After the first extraction, the remaining residues were subjected to cutin extraction at the following conditions (ethanol 40 wt%, 58 W, 100 % amplitude, 2 W/cm^2^, 17 min, 1/80 g/mL, pH 2.5). Watermelon showed the highest cutin level (55 %), while tomato and apple had 25 and 40 %, respectively [54]. The UAE was applied for 5-, 10-, and 15-min extraction time, amplitude 40, 70, and 100 % with pulse cycle 0.4, 0.7, and 1 s for polyphenolic compounds extraction from seed, pulp, and columella of soursop fruit. The optimal conditions for maximum polyphenol extraction were dependent on the type of raw material, such as peel resulted in 187.32 mg/g DM, columella 164.14 mg/g DM, pulp 33.24 mg/g DM, and seed 36.15 mg/g DM. The variation resulted in contents due to the matrix complexity of the matrix [144]. Peel and columella showed a higher yield of polyphenolic compounds (32–37 %) as compared to conventional processing for 2 h (14–16 %) [50]. US extracted Anthocyanin and antioxidants from sweet cherries' skin at the following conditions (ethanol 70 %, 40 kHz, 100 W, 40 ˚C, and 30 min). The findings showed that US improved anthocyanin (14.48 ± 1.17 mg cyanidin 3-glucoside/100 g DW) and antioxidant activity (85.37 ± 1.18 μMTrolox/100 g DW) [79]. Culinary banana dietary fiber was extracted by alkaline and UAE in combination with alkaline extraction. The optimum conditions were 25 mL/g solute, 49.48 % amplitude, temperature 80 °C, and time 10 min. The highest yield and TDF were 71.05 % and 83.38 g/100 g, obtained by the US and alkaline treatment combination. Moreover, regular honeycomb structure, maximum crystallinity (25.86 %), fine particle size, and thermal stability were observed in combined treatment. The overall finding suggested that the US, in combination with alkaline, was promising for extraction [77]. Date waste is high in moisture and organic compounds, which lead directly to environmental pollution. On the other hand, the dates processing industry is a rich source of sugars, fibers, proteins, and vitamins, which could be used in various bioprocesses [145]. Bioactive compounds were extracted from date seeds by NADESs in combination with UAE. The optimum extraction conditions were US amplitude of 90 %, extraction time of 25 min, NADES content of 70 %, and solid-to-liquid ratio of 1:30 g/mL. TPC and DDPH at optimum conditions were 145.54 ± 1.54 (mg GAE/g powder) and 719.19 ± 2.09 (mmol TE/g powder), respectively. This research demonstrated that UAE, in combination with NADESs, extracted higher amounts of phenolic components, which could be applied in the food, pharmaceutical, and cosmetic industries [46]. The UAE was carried out to assess the phenolic compounds of artichoke by-products. The optimum conditions were temperature: 60 °C, time: 60 min, solvent: 50 % ethanol: water. The outcomes showed TPC (22.4 ± 0.2 mg GAE g^-1^dw), which mainly included dicaffeoylquinic acid (32.8 ± 0.6 mg CAE g^-1^dw) and chlorogenic acid (14.1 ± 0.2 mg CAE g^-1^dw) [104]. Red beetroot is famous for higher betalains, red pigment, polyphenols, fiber, and nitrate. Beetroot juice is high in demand, leaving much waste behind. Betalains and polyphenols were recovered from whole dried beetroot juice waste. The US was applied at the following conditions: 44 KHz, 30 min, 30 °C, 35 W. Ethanol, and water mixture were proven more effective than single solvents. The UAE was effective in the recovery of betalains and polyphenol compounds from dried pulp. The total betalains and polyphenols were assessed for antioxidant capacities during four weeks of storage. Betalains showed degradation at room temperature compared to −20 °C, while polyphenols were less affected by temperature. The total betalains extractability with 30 v/v ethanol was 7.7 %, and polyphenols recovery was 6.86 ± 0.23 mg/g at 30 % v/v ethanol. The overall finding concluded that dried pulp waste from the beetroot juice industry demonstrated good betalains and polyphenols content [107]. Dietary fiber and phytochemicals were extracted from bottle gourd seeds by alkaline, enzymatic, and US-assisted alkali extraction. The US at the following optimized conditions LS; 1:23.90 mg/mL, time; 27.20 min and amplitude; 47.76 % resulted in 75.81 % dietary fiber. The US-assisted alkaline extraction at amplitude 70 %, temperature 45 °C, and 15 min time duration gave a higher phytochemicals yield of 8.24 %. While TPC (55.96 ± 0.75 mg/g), TFC (14.36 ± 0.45 mg/g), and antioxidant activity (35.08 ± 0.15 %) were improved as well [93]. In conclusion, the US is a suitable green technique for extracting bioactive compounds from fruits and vegetable waste for food, nutraceuticals, and medical field applications.

### 3.5 Lycopene, carotenoids, and related antioxidants

The US was employed on orange peel waste for bioactive compound extraction. The findings suggested that the US at optimum conditions (400 W, 30 min, 50 % ethanol in water) resulted in total carotenoids (TCC) (0.63 mg ß-carotene/100 g), vitamin C (53.78 mg ascorbic acid/100 g), and polyphenols (105.96 mg GAE/100 g). The major TPC in all orange peel samples was hesperidin (113.03 ± 0.08 mg/100 g) [44]. Wang and coworkers worked on mandarin orange peel and extracted phytochemicals such as tangerine and nobiletin by UAE at 85 % LS ratio 20:1 (mL/g), 40 min, 50 °C, powder mesh size 100, and 150 W. The outcomes showed that UAE increased the yield 1.5 times compared to simple solvent extraction [38]. The US was applied on the citrus peel for extraction efficiency. The UAE decreased extraction time to 20 min compared to the conventional process of 185 min. In UAE, the maximum d-limonene yield (32.9 mg/g, 97 %) was obtained by using hexane as a solvent at the following conditions: biomass: solvent (1:10), agitation speed (300 rpm), temperature (60 °C), ultrasonic power (80 W), 50 % duty cycle and frequency 25 kHz. This could suggest that UAE is an efficient and economical method for d-limonene extraction from fresh sweet lime peel [65]. Orange peel β-carotene pigment, in combination with enzyme and ethanol as solvent, was extracted. The optimum conditions were a pectinase concentration of 0.40 % (w/w), time 115.55 min, and pH 5.11. At the following processing conditions, the maximum content of β-carotene (209.14 ± 0.40 ppm), antioxidant activity (91.23 ± 0.40 %), and color parameters (L* 15.22 ± 0.03, a* 3.76 ± 0.21, b* 8.31 ± 0.01) were obtained. The results showed that US processing is effective for pigment extraction [66]. Orange peel carotenoids with olive oil as a solvent were extracted. The outcomes showed that carotenoids achieved values of 1.85 and 1.83 mg/100 g DW at optimum conditions of 35 min, 42 °C, and an LS ratio of 15 mL/g [42]. They were subjected to US for antioxidant extraction at 30 min, 60 ˚C, and an LS ratio of 15 mL/g. The results showed that gallic acid had the highest value (157.08 mg/100 g DW), whereas the lowest value was found for ferulic acid (20.91 mg/100g DW), and total antioxidant capacity was 2.79 g GAE/100g DW [43]. The US was applied to determine the bioaccessibility of carotenoids in an in vitro digestion model at the amplitude of 30 %, power 400 W, and frequency 24 kHz from mango peel and pulp. The UAE peel bioactive compound percentage was improved by β-cryptoxanthin (46.93 %), lutein (35.21 %), and β carotene (32.62 %), while in pulp β cryptoxanthin, β carotene, and lutein improved by 44.16, 44.01 and 46.04 % as compared to control-paste. In control-peel, non-bio-accessible β-carotene content was higher (79.48 %) than in UAE samples. The US improved bioaccessibility in vitro digestion in mango by-products [83]. Peach palm peel is a rich source of carotenoids. HPLC-DAD investigated the impact of different extraction methods on the carotenoid profile. Extraction was done by maceration (acetone & ethanol), shaker, magnetic stirring, and US with ethanol. The outcomes demonstrated that UAE gave maximum TCC (67 mg/100g), whereas maceration resulted in acetone and ethanol (63 and 52 mg/100g). The last extraction was shown in magnetic stirring and shake extractions (44 mg/100g). HPLC-DAD data revealed that the carotenoid profile remained unchanged regardless of the extraction method. However, the z isomer β-carotene in shaking was 18 %, US 17 %, and magnetic stirring 15 %, while maceration was 7 and 8 % with acetone and ethanol, respectively. This study suggested that the US, as a safe technology, could be used for carotenoid extraction [86]. The US and MW were applied on pumpkin peel for carotenoid extraction using corn and olive oil as solvents. The TCC ranged between 26.91 ± 1.96 and 34.35 ± 0.94 mg/100 g, higher than the conventional technique (22.24 ± 1.03 mg/100 g). This yield increase by applying these technologies is either by processing temperature, US, MW penetration depth, or carotenoids' solubility in edible oils. The UAE with olive oil is more effective for major carotenoid extraction (lycopene 87.0; β–carotene 18.7 mg/L). The DDPH inhibition percentage in US and MW-treated samples was 92.49 ± 1.84 and 94.16 ± 1.06 %, respectively, while in the conventional method was 58.57 ± 2.34 % [146].

The US at 42 kHz, 240 W, 60 °C, 60 min, and LS ratio 0.0004 g/mL was applied on mandarin epicarp for carotenoid extraction. These treatment conditions yielded the highest TCC (140.70 ± 2.66 mg β-carotene/100 g of DW). Moreover, variable variations such as a decrease in LS ratio and an enhancement in temperature caused the increase in TCC extraction [78]. This increase in extraction may be attributed to solvent diffusion in the matrix and an increase in carotenoid transfer to the environment [147]. The US was applied on tomato waste to recover lycopene content at 50 Hz, 45 min. Lab-prepared tomato waste has higher lycopene content (57.87 ± 5.30 μg/g fresh wt.) as compared to industrial (27.11 ± 0.83 μg/g fresh wt.). The average lycopene amounts by various techniques were observed in the US (45.51 ± 1.84 %), freeze-drying (104.10 ± 1.23 %), and US + freeze drying (138.82 ± 6.64 μg/g fresh wt). Freeze drying and US improved lycopene content by 2.8 and 0.68 folds, and their combined treatment enhanced industrial tomato waste yield by 4.12 folds. Freeze drying ruptures the cell structure, facilitates US processing, and enhances its extraction ability [114]. The US was applied to hot pepper pulp for carotenoid and capsaicinoid extraction. The optimum conditions were amplitude 60 % at 60 °C for 5 min. The US-treated sample outcomes were compared with the conventional method and eight h maceration. The UAE showed 235.46 mg β-carotene/100 g and 1148 μg/g capsaicin content. It was a more effective treatment due to higher extraction and short duration [148].

The US was applied for carotenoid extraction from carrot pomace. The optimum conditions for the US were extraction time (17 min), temperature (32 ˚C), and ethanol concentration (51 %), which resulted in the highest (31.82 ± 0.55) total carotenoid content. At the same time, 16 min extraction time, 29 °C temperature, and 59 % ethanol concentration resulted in a mixture of lutein (5.77 ± 0.19), lycopene (2.65 ± 0.12), and β-carotene (14.89 ± 0.40). Overall, the findings suggested that the US is a promising technique for carotenoid extraction from carrot pomace compared to conventional methods [110]. The UAE was carried out for carotenoid extraction from cantaloupe waste. The main carotenoids obtained were lutein (63.24 ± 0.73 μg βCE/g dw) and β-carotene (56.43 ± 0.11 μg βCE/g dw). The outcomes suggested that 100 % amplitude, 10 min extraction time, and 80 % hexane solvent 55 mL/g LS ratio were optimum treatment conditions. The 124.61 ± 3.82 μg/g carotenoid content was obtained at these processing conditions. The findings concluded that the US is an effective technology for carotenoid recovery from cantaloupe waste [102]. The US was assessed for ethanolic antioxidant extract extraction from lettuce waste at 400 power, 24 kHz frequency, and 120 s treatment duration. It gave 81 μg/mL polyphenol yield and 101 μg TE/mL antioxidant capacity, significantly higher than SLE at 50 °C and 15 min [111]. Defatted bitter seeds were subjected to pulsed US for protein extraction. The US at the following conditions was applied on the following power (300, 375, and 450 W) and time (2.50, 5.00, 7.50, 10.00, 12.50, 15.00, 17.50, and 20.0 min) to investigate the effects on protein yield and quality characteristics. The yields were higher at 375 W (31.05 %), followed by 450 W (28.93 %) and 300 W (23.79 %). With the increase in power level, yields decreased gradually due to protein aggregation. The water holding capacity (2.76 g/g), emulsion capacity (68.66 %), and emulsion stability (36.40 %) were higher than in conventional extraction [103]. All the above studies proved that the UAE is an effective approach to recovering the bioactive compounds from fruit and vegetable waste.

## 4 Recent upcycling approaches

### 4.1 Upcycling of waste for food product development

Food processing industry waste is a rich source of bioactive compounds. Due to the unique flavor, color, and nutritional value of fruits are incorporated into different items such as confectionery and baking, leading to effective waste utilization [149]. Factors such as fruit variety, ripening stage, and post-harvest handling conditions could influence the by-products' nutritional content. In addition, functional ingredient preparation greatly impacted the quality and quantity of functional ingredients. All of these features should be undertaken to maximize the nutraceutical potential of enriched products [150]. Table 3 describes the valorization of fruit and vegetable waste in functional food development.

**Table 3:** Applications of obtained biomolecules in functional foods.

**Food source**	**Function**	**Amount**	**Target nutrient**	**Functional food**	**Key findings**	**Reference**
Pumpkin peel	Fortification	15/100 g/mL	β-Carotene	Mayonnaise	β-carotene 127.93 mg/100 g DM, lecithin 0.098 %, polyglycerol 1.902 %.	[153]
Apple pomace	Fortification	4 and 8 %	Dietary fiber	Beef burgers	Aw 0.912, plate count agar 7.5 CFU/g.	[75]
Sweet cherries skins	Enrichment	5 % & 10 %	Anthocyanins	Yoghurt & marshmallows	Yoghurt: Reduced antioxidant activity ∼ 32 % and ∼ 29 %, No anthocyanins. Marshmallows: Anthocyanin 1.8 mg c_3_ G/g DW, antioxidant activity 21 %.	[79]
Mandarin epicarp	Natural colorant	20 g	Carotenoids	Cake & bread	Bread: β- carotene 4.21, α- carotene 4.00, β-cryptoxanthin 5.02, Zeaxanthin 4.98, lycopene 2.74, ΔE crust 15.90, ΔE crumb 2.15 Cake: β- carotene 2.52, α- carotene 1.49, β-cryptoxanthin 2.04, Zeaxanthin 2.02, Lycopene 2.30, ΔE crust 15.91, ΔE crumb 2.81.	[78]
Bottle gourd seeds	Enrichment	3 % & 1 %	Dietary fiber & phytochemicals	Cookies	Appearance 8, taste 8.07, texture 7.92, overall acceptability 8.15.	[93]

The UAE and maceration used sunflower oil to extract β-carotene from pumpkins to prepare β-carotene-rich sunflower oil-fortified Mayonnaise. At 15/100 g/mL LS ratio, the different values of β-Carotene contents were detected. In the case of UAE (127.93 mg/100 g DM) and microemulsion (149.71 mg/100 g DM) with lecithin 0.098 % and polyglycerol 1.902 %, better results appeared than maceration + sunflower oil (99.83 mg/100 g DM) and maceration + n-hexane (125.75 mg/100 g DM). Mayonnaise formed with extracted β-carotene-rich sunflower oil had better sensory quality than control; however, during storage, it was more resistant to oxidation [151]. The apple pomace with ethanol (50:50, v/v) in 1:10 LS ratio was subjected to an ultrasonic bath at 45 ˚C for 45 min for bioactive compounds recovery. Fresh pomace yield increased twice from 7.12 % to 13.61 %, while UAE, in the case of freeze-dried pomace, showed a further increase in yield (from 21.64 % to 58.09 %) and TPC up to 10.05 mg GAE/g DW. Apple pomace freeze-dried powder with 40.19 % dietary fiber was utilized to fortify beef burgers (4 % and 8 %). The sensory evaluation gave better results for fortified burgers than for control. The water activity values were for beef burgers with 0 % apple pomace (0.952), for 4 % fortified (0.948), and for 8 % fortified burgers (0.943) after 8 days. Plate count agar for 4 % and 8 % burgers were 7.25 and 6.90 log CFU/g after 8 days [75].

The US at 42 kHz, 240 W, 60 °C, 60 min, and LS ratio 0.0004 g/mL was applied on mandarin epicarp for carotenoid extraction. These treatment conditions yielded the highest TCC (140.70 ± 2.66 mg β-carotene/100 g of DW). The extracted TCC was added as a natural colorant in bread and cake to lower tartrazine usage. The outcomes showed that cake has lower TCC than bread due to a higher baking temperature (215 °C) than bread baking temperature (180 °C). Bread contained carotenoids β-carotene (4.21), α-carotene (4.00), β-cryptoxanthin (5.02), zeaxanthin (4.98), and lycopene (2.74). In cake, β-carotene, α- carotene, β-cryptoxanthin, zeaxanthin, and lycopene were 2.25, 1.49, 2.04, 2.03, and 2.30, respectively [78]. Besides, the US extracted anthocyanin and antioxidants from sweet cherry skin at the following conditions (ethanol 70 %, 40 kHz, 100 W, 40 ˚C, and 30 min). The findings showed that US improved anthocyanin (14.48 ± 1.17 mg cyanidin 3-glucoside/100 g DW) and antioxidant activity (85.37 ± 1.18 μMTrolox/100 g DW). The extracted compounds were encapsulated via freeze-drying. This powder was incorporated into yogurt and marshmallows. These samples were analyzed for TAC and antioxidant activity at 4 °C for 7 and 21 days. In yogurt samples, antioxidant activity was reduced to 32 % and 29 %, and TAC was not detectable after 21 days. In marshmallows, a three-time increase in total anthocyanin content (1.8 mg c_3_ G/g) was observed, while antioxidant activity decreased by 13 % and 21 % in samples. It was noted that *Lacticaseibacillus casei* in the control group decreased by 54 % by 70 % in the 5 % powder addition product [79]. Bottle gourd seeds were used for dietary fiber extraction and then incorporated into cookies. Dietary fiber was extracted using alkaline, enzymatic, and UAE. The US treatment at optimum conditions' amplitude of 47.76 % and time of 20 min resulted in a higher yield of 75.81 %. These fiber-enriched cookies were prepared with 5 different formulations of dietary fiber (3, 6, 9, 12, and 15 %) and phytochemical beads (1, 1.5, 2, 2.5, and 3 %). The average sensory score was obtained at the following (3 % DF, 1 % phytochemical) for appearance (8), taste (8.07), texture (7.92), and overall acceptability (8.15). This research demonstrated that bottled gourd seeds can have applications as dietary fiber sources [93]. Therefore, it can be concluded that fruit and vegetable waste could make functional food that can used against specific diseases and benefit human health.

### 4.2 Upcycling of waste for food packaging and preservation

Food packaging is a part of food processing for its storage, transport, and distribution and ends at the consumer level. It is a tool utilized in food processing to protect the product from environmental stress. The main objective of food packaging is the protection of food commodities from external impacts (light, heat, and oxygen) and the preservation of nutrients. Moreover, it also aids in preventing tempering [152].

#### 4.2.1 Edible coatings and biopolymer films

Edible coating gained attention due to its biodegradable nature and potential to increase the shelf life of products [153]. Biopolymers based on fruits and vegetables have been extensively used in biodegradable film preparation, which provides preservation, longer shelf life, and sensory properties [154]. Various plant and animal source biopolymers like lipids, proteins, polysaccharides, and bioplastics are synthesized by microbes to develop eco-friendly packaging material that can potentially preserve functional components of food products [155].

The potato processing industry produces a vast amount of waste, which contributed to the circular economy approach. In addition, sweet lime pulp generated pomace after juice extraction, which is considered zero-valued and ineffective. However, biodegradable packaging films could consume this zero-valued waste in food packaging to reduce environmental stress. Composite films were produced by using potato peel powder and sweet lime pulp pomace in different proportions (0:1, 0.5:1, 1:1, 1:0.5, 1:0) with US treatment (45 min) and (0:1, 0.5:1, 1:1, 1:0.5, 1:0) with 60 min. The US applied a film solution at different processing times to break polymers into small pieces to create a film. All the films were investigated for their barrier and mechanical properties. The outcomes showed that increased processing time provided good film and potato peel powder favorable for film formation. So, the films with 0.5:1 with potato peel and sweet lime pulp proportion 0.5:1 were best. The other properties of films with 0.5:1 were water vapor permeability (7.25 × 10–9 g/Pa h m), moisture absorption (12.88 ± 0.348 %), water solubility (38.92 ± 0.702 %), breakage strength (242.01 ± 3.074 g) and elongation capacity (7.61 ± 0.824 mm). The thermal decomposition temperature of films with 0.5:1 was above 200 °C. Moreover, these films were tested for bread as packaging for 5 days with 1.5 % clove essential oil and successfully reduced weight loss, hardness, and surface microbial load [156].

**Fig. 3:**
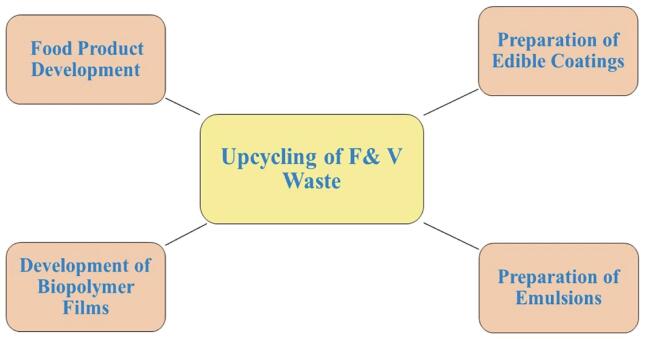
Upcycling of fruits and vegetables waste by the application of US.

Apple polyphenols and sodium alginate were used as base materials, and glycerol was used as a plasticizer to produce an antibacterial and anti-oxidation composite film by casting. The US-assisted electrospray method was applied for silver nanoparticle deposition in films. Sodium alginate and silver particle ratio in films at different ultrasonic times were assessed on sample films' mechanical, optical, and hydrophilicity. The outcomes suggested that sodium alginate and silver ration of 7:3 and processing US of 30 min was best for all properties. These films had good strength, softness, and low water vapor permeability (0.75 × 10–11 g/msPa). The composite films demonstrated a wide range of antibacterial activity, and *Escherichia coli*'s (92.01 %) antibacterial activity was higher than *Staphylococcus aureus's* (91.26 %). Adding apple polyphenols resulted in higher antioxidant activity (98.39 %). In a strawberry model, these composite films enhanced the shelf life for 8 days at refrigeration temperature compared to polyethylene films [157]. Upcycling of fruits and vegetable waste by applying ultrasound technique is described in Fig. 3.

## 5 Ultrasound technology for the treatment of beverage industrial wastewater

The fruits and vegetables processing industries generate large amounts of wastewater that contains a wide variety of organic compounds and showed 5 days of biochemical oxygen demand (500 to 6100 mg/L) and chemical oxygen demand (806 to 7732 mg/L), pointing to serious environmental concerns [24]. This wastewater treatment involves high-cost energy; hence, removing bioactive compounds is a serious challenge. This wastewater includes many polysaccharides, pigments, polyphenols, proteins, and fibers [158]. The US is an effective method for removing toxic and hazardous organic components from wastewater. Its application causes irreversible changes in the medium involving biological and chemical phenomena such as coagulation, oxidation, and depolymerization [159]. Besides these changes, the mechanical effect on the medium was also observed, including structure changes by US vibrational, which rely on the frequency and amplitude of waves. The US utilization in wastewater treatments helps remove toxic and resistant pollutants such as aromatics, surfactants, and dyes. This process involves the oxidative breakdown of resistant wastewater compounds [25].

Increasing coke production in consequent wastewater is a serious environmental issue. This wastewater includes large amounts of toxic compounds; therefore, coke wastewater treatment requires a combined processing of physicochemical and biological methods. The US was applied for coke wastewater treatment in a sequencing batch reactor. The researchers aimed to determine optimum conditions (amplitude and time) and wastewater treatment in a sequencing batch reactor. The following conditions were applied: amplitudes (31, 61.5, 92 & 123 μm) and times (120, 240, 480 & 960 s).The second aim involved two reactors (reactor A- coke wastewater in different proportions: 5, 10, and 20 %; reactor B- Sonicated coke wastewater in different proportions: 5, 10, and 20 %). The processing efficiency was determined based on the following factors: chemical oxygen demand, total organic carbon, ammoniacal nitrogen, and pH alterations. This research showed that US treatment enhanced the biodegradability of wastewater and decreased toxicity. The US treatment prior to biological treatment resulted in 10 % removal of selected parameters. The coke wastewater volume during processing significantly impacted results; 10 % of the coke wastewater mixture was allowed in the US field [160]. In conclusion, the US technology is effectively utilized to treat beverage industrial wastewater.

## 6 Limitations and future challenges

Apart from identifying bioactive compounds and safety risks, challenges also involve large amounts of solvent, long time for extraction, high temperature, and power of the US, which are some usual drawbacks linked with the extraction of compounds from natural food by-products [129]. The UAE is inefficient for heat-liable and oxidation-sensitive bioactive compounds. Limitations associated with by-product extracts are instability, less water solubility, and low bioavailability of components that restrict their application in the food industry [161]. For instance, citrus flavonoids have poor bioavailability and high sensitivity towards environmental conditions, which limits their use in industry as a food ingredient. Conversely, with technological advancement, there is a need to explore more approaches toward effectively utilizing waste and its safety assessment [34]. Its application in food systems requires special attention for extracted components' stability, including bioactive compounds. Besides US benefits in food processing, the cavitation process and temperature could produce free radicles, which cause degradation issues. It could also cause severe molecule configuration changes and cell wall component degradation, releasing enzymes that oxidize phenolics [162]. Cruz et al. [163] demonstrated that grape skin is sensitive to US processing. The TPC ranged between 18.4 and 31.0 %. Bioactive stability should be controlled to increase the positive effects of US processing in the food industry.

Besides the effective utilization of waste, there is also a need for wide research on the safety aspect of these by-products. Recycling generates various value-added products, but its introduction has risks in the food chain. Contamination is a basic challenge of transforming by-products into food products [164]. Moreover, by-products are novel food products with specific functions; hence, safe food use must be properly documented. This raises the need for more research on the safe use of by-products in the food industry [163]. In addition, the bioactive compounds in fruits in food require legal assessment and dose recommendations for safety issues. For these reasons, much research was done on the safety assessment of citrus-based compounds. However, there is still a need to explore the risk related to each extracted compound from waste to improve its application in the food industry. It provides advantages in terms of time, temperature, energy, and chemical requirements during the extraction of the bioactive compounds from fruits and vegetables by-products; however, the complete removal of chemical solvents with reasonable yield needs to be explored. The UAE process, in combination with other non-thermal techniques such as MW, PEF, and enzyme-assisted extraction of bioactive compounds from fruit and vegetable waste, requires more research. Moreover, the equipment’s development is needed to avoid direct contact with US horns.

## 7 Conclusion

Waste from the fruit and vegetable industry is a rich source of bioactive compounds and finds extensive applications across various industrial sectors. Due to their nutrient-dense and diverse composition, these wastes offer numerous health benefits. Consequently, they serve as an excellent source of natural ingredients for functional foods and food packaging. The potential to repurpose fruit and vegetable waste underscores the importance of recycling in boosting productivity and minimizing environmental impact. Ultrasound technology, an eco-friendlier alternative to traditional physical and chemical methods, effectively extracts valuable compounds from food waste and treats wastewater, enjoying high consumer acceptance. Adopting this approach can yield both economic and environmental benefits, transforming this sector into a sustainable, eco-friendly production system and promoting a circular, sustainable economy. It's crucial to evaluate fruit and vegetable waste for the recovery and characterization of bioactive compounds, and to explore their use in chemicals, nutraceuticals, and as food ingredients. Additionally, thorough research into the safety aspects of introducing these waste-derived products into the food chain is essential to enhance their applications in the food industry.

## CRediT authorship contribution statement

**Brera Ghulam Nabi:** Conceptualization, Methodology, Writing – original draft. **Kinza Mukhtar:** Conceptualization, Methodology, Writing – original draft, Writing – review & editing. **Sadia Ansar:** Writing – review & editing. **Syed Ali Hassan:** Methodology, Data curation, Writing – review & editing. **Muhammad Adnan Hafeez:** Methodology, Data curation, Writing – review & editing. **Zuhaib F. Bhat:** Methodology, Data curation, Writing – review & editing. **Amin Mousavi Khaneghah:** Conceptualization, Methodology, Project administration, Resources, Supervision, Validation, Visualization, Writing – original draft, Writing – review & editing. **Ahsan Ul Haq:** Methodology, Data curation, Writing – review & editing. **Rana Muhammad Aadil:** Conceptualization, Writing – review & editing, Supervision.

## Declaration of competing interest

The authors declare that they have no known competing financial interests or personal relationships that could have appeared to influence the work reported in this paper.
